# Four-point impedance as a biomarker for bleeding during cochlear implantation

**DOI:** 10.1038/s41598-019-56253-w

**Published:** 2020-02-17

**Authors:** Christofer Bester, Tayla Razmovski, Aaron Collins, Olivia Mejia, Søren Foghsgaard, Alistair Mitchell-Innes, Chanan Shaul, Luke Campbell, Hayden Eastwood, Stephen O’Leary

**Affiliations:** 1 Department Otolaryngology, University of Melbourne, Victoria, Australia; 2 Royal Victorian Eye and Ear Hospital, Victoria, Australia; 3 sENTro Head and Neck Clinic, Manila, Philippines; 4 Dept ORL Head and Neck Surgery, Cohenhagen University Hospital Rigshospitalet, Copenhagen, Denmark; 5 1Musgrove Park Hospital, Taunton, Somerset UK; 6 Shaary Zedek Medical Centre affiliated with the Hebrew University School of Medicine, Jerusalem, Israel

**Keywords:** Biomarkers, Translational research

## Abstract

Cochlear implantation has successfully restored the perception of hearing for nearly 200 thousand profoundly deaf adults and children. More recently, implant candidature has expanded to include those with considerable natural hearing which, when preserved, provides an improved hearing experience in noisy environments. But more than half of these patients lose this natural hearing soon after implantation. To reduce this burden, biosensing technologies are emerging that provide feedback on the quality of surgery. Here we report clinical findings on a new intra-operative measurement of electrical impedance (4-point impedance) which, when elevated, is associated with high rates of post-operative hearing loss and vestibular dysfunction. *In vivo* and *in vitro* data presented suggest that elevated 4-point impedance is likely due to the presence of blood within the cochlea rather than its geometry. Four-point impedance is a new marker for the detection of cochlear injury causing bleeding, that may be incorporated into intraoperative monitoring protocols during CI surgery.

## Introduction

The preservation of cochlear structure and residual functional hearing has become the standard of care for cochlear implantation (CI). Hearing preservation is important to facilitate combined electrical and acoustic stimulation of the cochlea, as this improves speech recognition in noise and music appreciation^1–4^. Cochlear structural preservation will ensure that the ear is ready for future, regenerative therapies^5,6^.

Structural and functional preservation of the cochlea depends not only upon the electrode design, but also the surgery. Electrodes must be introduced into the cochlea without causing injury. Until recently, technologies have not existed to guide the surgeon during the implant procedure; the operation has been conducted “blind” without the provision of feedback. Over recent years, we and others have begun to monitor cochlear function during cochlear implantation^7–10^, using the CI’s own electrodes to monitor the electrophysiological response of the ear to acoustic stimulation. This technique, known as electrocochleography, has provided valuable information to guide surgeons during the operation; if the electrophysiological response is preserved during surgery, residual hearing is better after implantation^7–10^.

**Figure 1:**
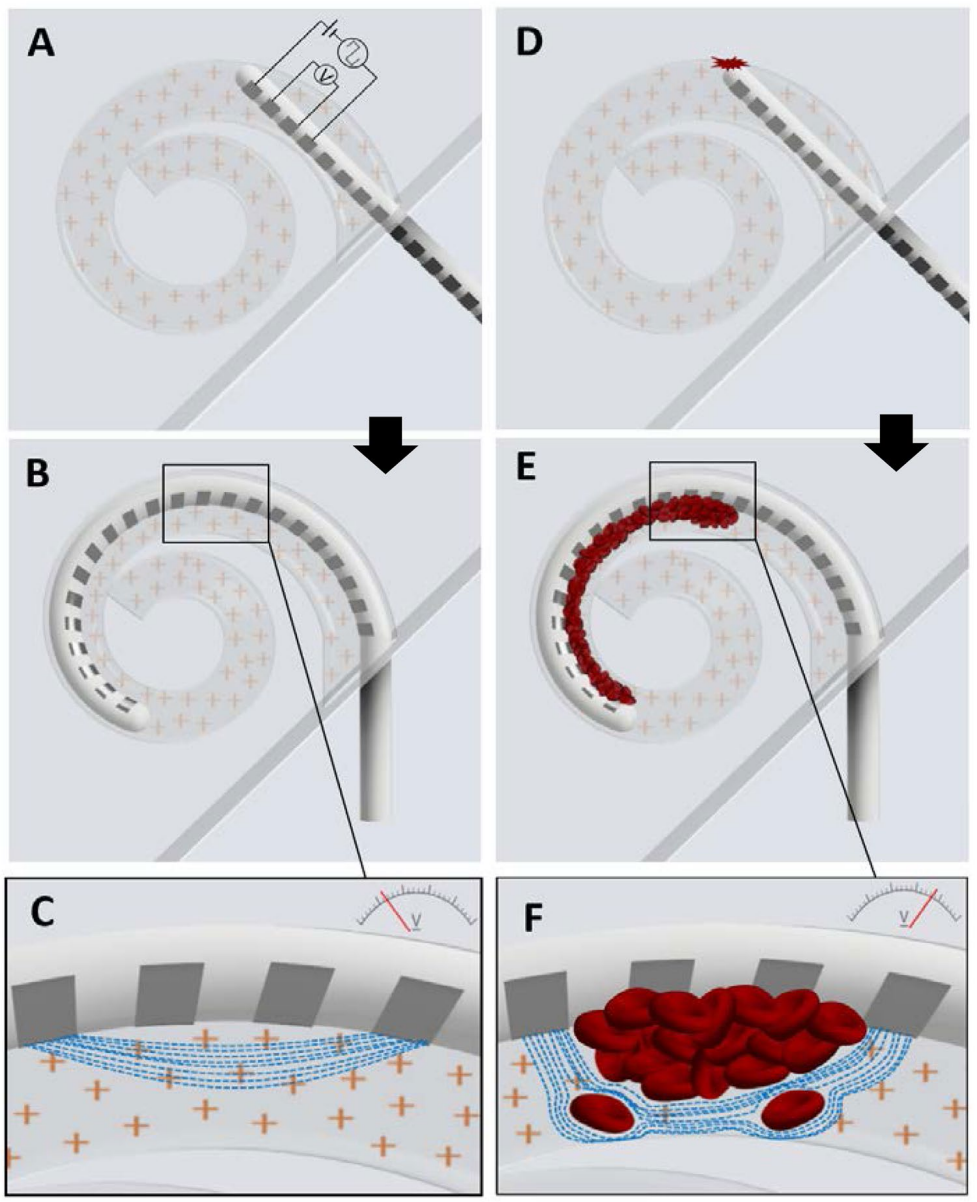
Illustrations of cochlear implant surgeries with/without trauma to the lateral wall and how the presence of blood cells effects impedance. (**A–C**) Illustration of a cochlear implant where lateral wall trauma did not occur. (**A**) Illustration of how four-point impedance is inferred using a cochlear implant. The current is supplied to the outer electrodes in the quartet and the voltage is measured between the two inner electrodes. This is repeated along the whole array, resulting in 19 quartets. (**B**) Insertion completed with the cochlea filled with perilymph. **C**) The current paths of the stimulus for a four-point impedance measurement through perilymph. The current has little resistance and therefore the voltage is low. (**D–F**) Illustration of a cochlear implant where lateral trauma occurred, resulting in the infiltration of blood. (**D**) Insertion of the cochlear implant where the electrode array comes into contact with the lateral wall and causes damage. (**E**) Cochlear implant completely inserted with blood pushed into the cochlea along the electrode array. (**F**) The current paths for the stimulus when blood is present in between the two inner electrodes. The current has more resistance since it does not pass through the cells and the reduction in available ions, resulting in a higher voltage.

This paper is motivated by a desire to increase the scope of intraoperative monitoring during CI surgery. Current methods allow real-time detection of cochlear dysfunction, but these do not assess cochlear injury directly. Here we report on a method that has this potential. We have monitored “four-point” electrical impedance (4PI) from the implant’s intracochlear electrodes during CI surgery. This impedance measurement is acquired by passing current between two outer electrodes whilst the voltage (from which the impedance may be inferred) is measured between two inner electrodes (Fig. 1A). The method is believed to assess the bulk impedance between the two inner electrodes, and has been used to differentiate between tissue and fluid types^11,12^ such as blood, urine, muscle, lung, fat, liver and spleen tissue, as well as pathological from normal tissue^13^. The principle upon which 4PI is believed to work is illustrated in Fig. 1, as it might apply to the cochlea. With a cochlear implant, current is conducted between source- and sink- electrodes via ions (mainly sodium and chloride) in the perilymph (Fig. 1A).

During its insertion, a straight CI electrode array will contact the outer wall of the cochlea and this can lead to endostial (or more severe) trauma. When the trauma causes bleeding (Fig. 1D,E), the volume of perilymph available to conduct current is effectively reduced by the presence of blood cells and the current will pass around the cells, not through them (Fig. 1F).

Consequently, there are fewer ions available to conduct current and this increases the electrode impedance. Because cochlear implants are designed to deliver constant current, the voltage required to drive electrons through the blood-filled perilymph must therefore increase (following Ohm’s Law). Four-point impedance is inferred from this voltage, which is measured from the inner-two electrodes. We were also cognoscente that cochlear shape and size might impact upon 4PI. The number of ions available to conduct current is inversely related to the volume of the conductive medium, so we might expect that 4PI will rise as a CI electrode is inserted deeper into the cochlea because the turn diameter decreases. Similarly, 4PI might be expected to be higher in a smaller cochlea. So, in order to interpret 4PI within the cochlea, the influence of both the presence of blood and cochlear anatomy, needs to be understood.

In this study clinical data are presented, where 4PI measurements are related to loss of residual hearing; the anticipated functional consequence of intracochlear bleeding^14,15^. To provide greater insights into the interpretation of these clinical data, real-time intraoperative monitoring of hearing was also undertaken during these surgeries. Next, the association between intracochlear bleeding and 4PI was tested directly in an animal experimental model of cochlear implantation. Finally, we undertook a series of *in-vitro* experiments to further strengthen the association between 4PI and bleeding, and to ascertain whether other factors that may have impacted upon 4PI, such as cochlear geometry and size, could have affected the clinical and experimental results presented here.

## Results

### Clinical studies

Fifty-one adults with residual acoustic hearing prior to surgery underwent cochlear implantation with a commercial device that had a flexible, straight half-banded 25 mm-length electrode array. Real-time intraoperative monitoring of hearing was undertaken during implantation, where electrocochleography (ECochG) was recorded directly from the tip electrode on the implant array, in response to a high intensity acoustic tone burst (500 Hz). Four-point impedance was measured immediately after the electrode array had been fully implanted. Next, the conventional common-ground impedance, measured by the commercial device’s software was acquired. The preservation of residual hearing was determined 3 months after implantation, and any new episodes of dizziness reported in the medical record were noted. The angular depth of the implanted electrode was determined by post-operative radiological imaging.

During the intra-operative monitoring of cochlear function with ECochG, the hair-cell derived cochlear microphonic (CM) potential was detected in all but 4 cases. In 37 patients a fluctuation or reduction in CM amplitude, of at least 30% relative to its greatest magnitude (a “CM drop”), was observed at some time during electrode insertion. In the other (10) patients, the CM amplitude steadily increased during electrode insertion. Three months after surgery, the median hearing loss (of the average hearing across 0.25, 0.5 and 1 kHz) was significantly lower for those patients that did not experience a drop in CM (16.67 dB, N = 10) than those that did (40 dB, N = 37; *X*^2^ = 4.13, *p* = 0.04). All 25 patients who lost all measurable hearing exhibited a CM drop during implantation.

**Figure 2:**
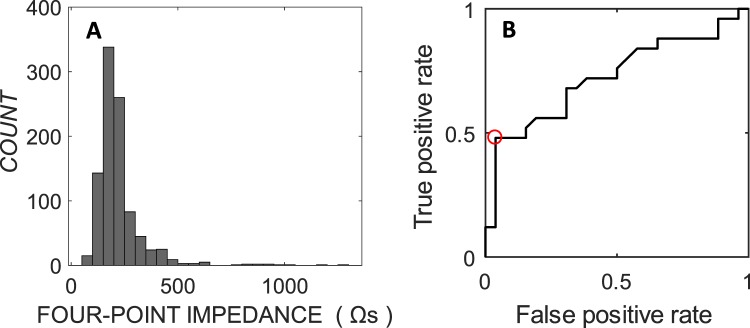
Four-point impedance distribution and Receiver Operator Curves, for four-point impedance and total hearing loss. (**A**) The distribution of four-point impedance immediately following electrode insertion in all 4-electrode measurements for all patients (969 measurements). (**B**) Receiver operator curve for the immediately-post insertion four-point impedance values predicting total hearing loss by 3-months post-op, point of maximum efficiency represented by red circle.

Four-point impedances were recorded immediately after implantation of the CI’s electrode array. The distribution of all impedance measurements is presented in Fig. 2A. To explore whether patients with higher 4PI were more likely to have lost their residual hearing, a receiver operator curve (ROC) was constructed. The maximum 4PI across each implant array was identified, and the sensitivity and specificity of detecting a total loss of hearing was calculated for different 4PI cut-offs. The area-under curve for ROC shown in Fig. 2B was 0.72 (95% CI calculated from N = 1000 bootstrap replicas from 0.54 to 0.85). The maximum efficiency (cross-over point of sensitivity and specificity) was at 387 Ω, returning a sensitivity of 95% (95% CIs from 81% to 100%) and specificity of 43% (95% CIs from 21% to 67%). Four-point impedances exceeding this level were deemed to be “high”, and thirteen patients met this criterion. These were not only likely to lose all of their residual hearing, also to experience dizziness in the perioperative period (9 or 13 with high 4PI, 9 of 38 without; *X*^2^ = 8.80, *p* = 0.003).

**Figure 3:**
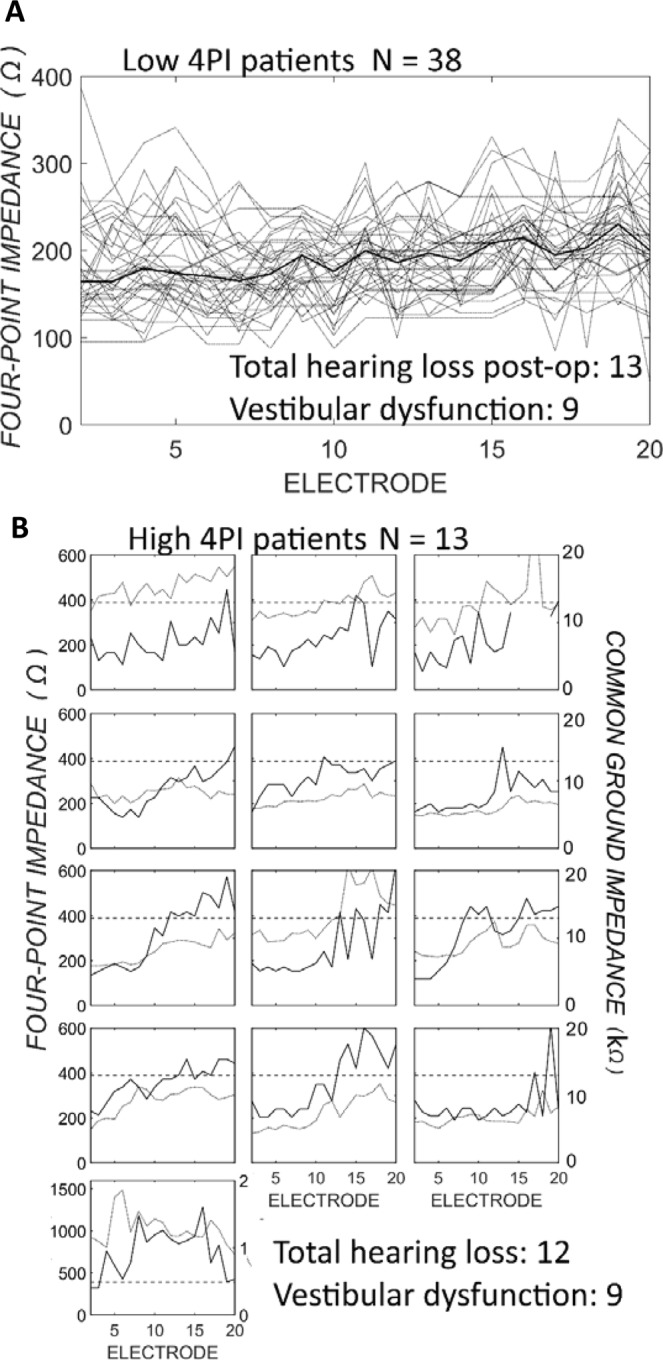
Clinical four-point impedance and common ground impedance immediately after implantation. (**A**) Four-point impedance values in 38 patients with ‘low’ four-point impedance, defined as impedances lower than those at the point of maximum efficiency for prediction of total hearing loss on the receiver operator curve (Fig. 2B). Individuals are presented as grey lines. Electrodes are numbered from base of the cochlea. (**B**) Individual four-point impedances (black line) and common ground impedance (grey line). The dashed line defines the low/elevated four-point impedance transition point, as determined by the ROC point of maximum efficiency (388 Ω). The numbers of patients with total hearing loss, and post-operative vestibular dysfunction in each group is mentioned in each sub-figure.

Four-point impedances were plotted across the length of the electrode array for individual patients in Fig. 3. In the patients with low 4PI across the electrode array (Fig. 3A) there was a gradual basal-to-apical rise in 4PI. Measurements from the apical 10 quartet of two stimulating, and 2 recording electrodes were on average 28 Ω higher than those from the basal ten (Fig. S1). In patients with one or more high 4PI measurement (Fig. 3B), the impedance was usually elevated on the apical electrodes (11/13). Patients with high 4PI on apical electrodes did not have deeper electrode insertions than the others, as determined by the angular insertion depth of the tip electrode on post-operative radiological imaging (medians of 378 and 400 degrees respectively, *X*^2^ = 2.08, *p* = 0.15).

Pre-operative audiometric thresholds were not significantly different between patients with elevated or lower 4PI (medians of 65 and 75-dB HL at 0.5-kHz, *p* = 0.35). Similarly, age at implantation, a demographic factor commonly associated with post-operative hearing preservation, was not significantly different between those with or without 4PI elevation (medians of 68 and 75 for the low 4PI and elevated 4PI respectively, *X*^2^ = 1.88 *p* = 0.17).

The common ground impedance derived from commercial software measured intraoperatively was significantly higher for the high 4PI patients than the low 4PI patients (mean across the array of 9.0 kΩ and 7.0 kΩ respectively, *X*^2^ = 6.4, *p* = 0.01). While the common ground impedance was greater when 4PI was high, it did not have predictive power for total hearing loss at 3 months after surgery (AUC of 0.54, 95% CI from 0.37 to 0.70).

In summary, high 4PI was associated with a high risk of losing residual hearing and experiencing dizziness by 3 months after CI surgery. These findings might be expected in the presence of intracochlear bleeding, which causes inflammation and a loss of inner ear function^14,15^ in animal models. To test the association between 4PI and bleeding more directly, experimental methods were employed.

### *In vivo* experiments

4PI measurements were made from 13 ears of 9 adult tricolour guinea pigs that had undergone cochlear implantation using a custom-built 4-banded cochlear implant. Implantation of the electrode array was performed via a small hole drilled into the basal turn of the cochlea (a “cochleostomy”). In 11 of the 13 ears, blood was injected into the cochleostomy after electrode insertion and baseline 4PI recordings. The other two procedures acted as controls, where 4PI was recorded for 2–3 minutes, but blood was not injected. The duration of recordings for the control group were shorter to minimize the likelihood of blood entering the cochlea from the cochleostomy and obscuring the results.

**Figure 4:**
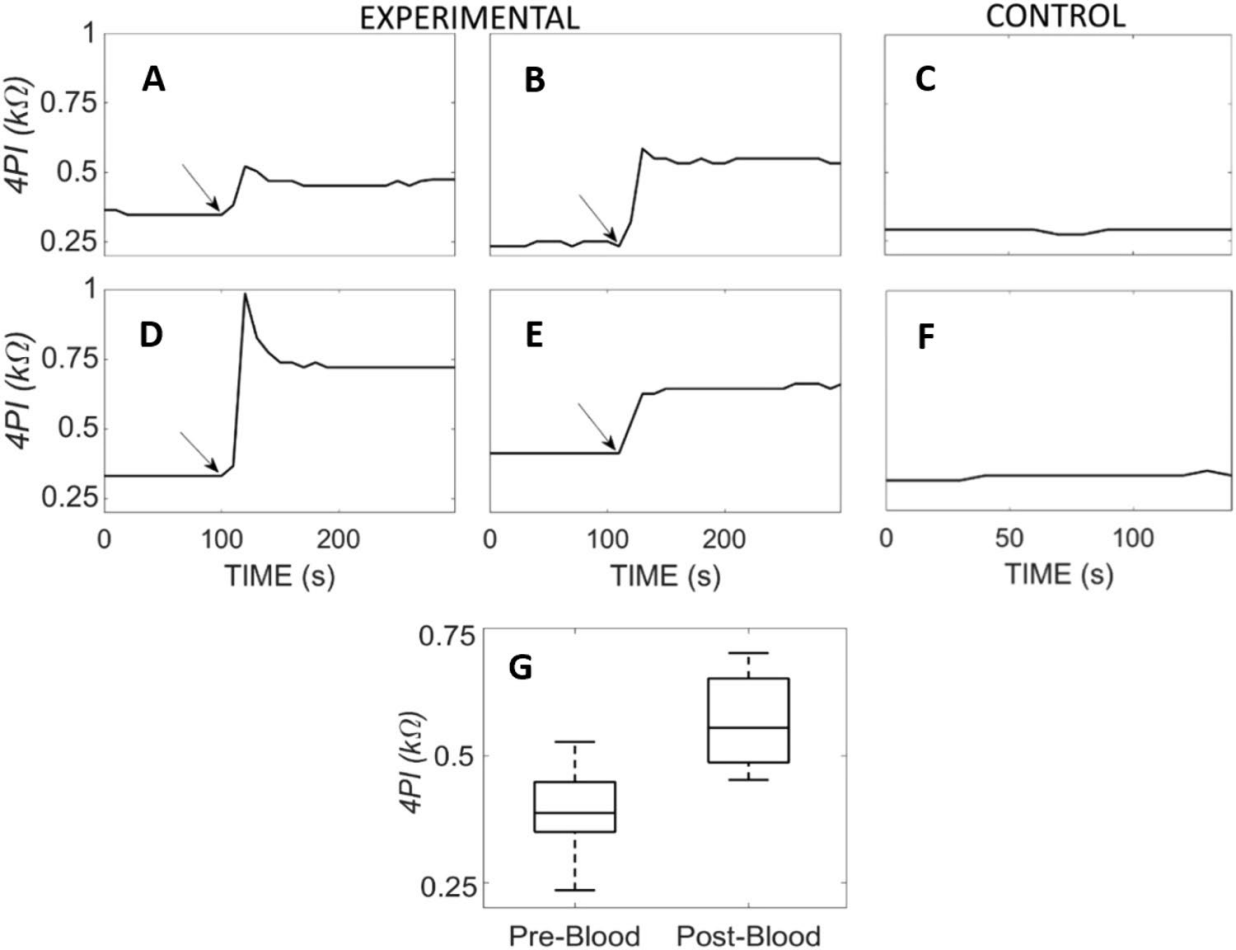
Experimental Four-point impedance and the introduction of blood into a cochlea. Four-point impedance recordings from 4 of the 11 procedures before and after blood was injected into the cochleostomy. The arrows indicate the time blood was injected. (**A**,**D**) show recordings from procedures that injected cold blood and (**B**,**E**) are recordings from procedures that blood at body temperature was injected. (**C**,**F**), the four-point impedance measurements from the two controlled procedures where blood was not injected into the inner ear. (**G**) A boxplot representation of the four-point impedance pre- and post- blood injection from 9 of 11 cases that were exposed to blood. 2 of 11 cases resulted in outliers are not shown.

**Figure 5:**
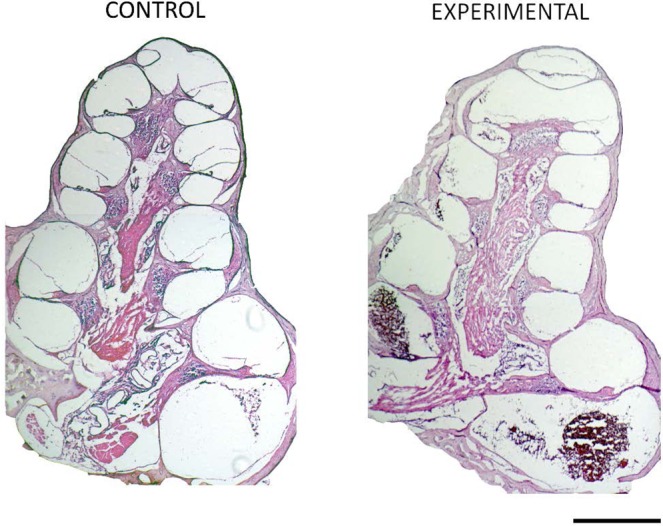
Experimental blood injection histology. Cross sections of a control cochlea (left) that received only a cochlea implant and an experimental cochlea (right) that was implanted and had blood injected into the basal turn. Scale bar for 500 µm.

Figure 4 shows 4PI measurements before, during and after the injection of blood in four experimental ears, and the two controls. Inspection with the operating microscope confirmed that blood was always present in scala tympani after injection. Histological analysis of the cochleae (example shown in Fig. 5), demonstrated that scala tympani of experimental cochleae contained significant quantities of clotted blood, but control cochleae exhibited occasional red blood cells only.

The 4PI values before blood injection averaged 379 ± 149, with a maximum of 528 Ω. After blood was injected in the cochlea, the mean 4PI increased to 586 ± 134 Ω, the maximum 1950 Ω. The mean increase was 190 ± 153 Ω (Fig. 4G) and ranged from 50-1500 Ω. Two of 11 cases exhibited a marginal impedance rise of 50 Ω, and these had a higher initial impedance of approximately 500 Ω.

In 8 of 11 cases, the blood injected into the cochleostomy was at body temperature. In these cases, 4PI exhibited a rapid rise that maintained its magnitude thereafter (Fig. 4B,C). Three of 11 ears received an injection of cold blood into the inner ear, and in these cases the plateau was preceded by a spike (Fig. 4A,D). In the control cases (Fig. 4C,F), the 4PI measurements remained stable throughout the recording at 292 and 327 Ω.

### *In-vitro* studies-geometry and cochlear size

The presence of an electrode array within a smaller-sized cochlear region, such as an upper turn of the cochlea, or in close proximity to the cochlear wall(s), the availability of electrically conducting ions may be reduced, and this would be expected to increase 4PI. To explore the extent to which these factors impacted upon 4PI, and the size of these effects relative to the presence of blood, *in vitro* experiments were undertaken in 3D printed models.

4PI measurements were made after clinical cochlear implant arrays (of the same make as those used in the clinical studies) had been implanted into 3D models of a human cochleae that had been printed in resin. In three experiments the electrode array was inserted with the electrical contacts facing towards the modiolus, which is the correct orientation for this implant (Fig. S2A,B). Four-point impedance increased gradually from the base to the apex of the cochlear model and was slightly higher in the 40 μL cochlear model (range: 308–520 Ω) than the 50 μL model (range: 281–482 Ω). In another three experiments the electrical contacts were facing the lateral cochlear wall, which emulates the “worst-case” scenario for incorrect electrode orientation during its implantation (Fig. S2C,D). The impedances rose in the mid-portion of the electrode array and then remained elevated once fully inserted, with greater elevation apparent in recordings derived from the 40 μL cochlear model. Consideration of this worst-case scenario was warranted because less-than-ideal electrode orientations were encountered in the clinical cohort; on 4 of 29 videos that were available for review, the electrode contacts were facing more towards the lateral, than the medial wall of the cochlea. Three of these cases had low 4PI, and high 4PI was observed in the other. (Videos from 22 patients with low 4PI and 7 from patients with high 4PI were reviewed).

Electrodes were also implanted into cylindrical volumes, where either the volume or the diameter was controlled, in order to better understand the impact of shape geometry and size upon 4PI measurements. When electrodes were inserted into straight cylindrical models of varying volume (30–80 µl) but fixed diameter (2.2 mm), volume had a small, negative effect on 4PI (Fig. S3A). When volume was fixed (60 µl), cylinder diameter was found to have had a greater effect on 4PI, and there was an inverse relationship between the two (Fig. S3B).

**Figure 6:**
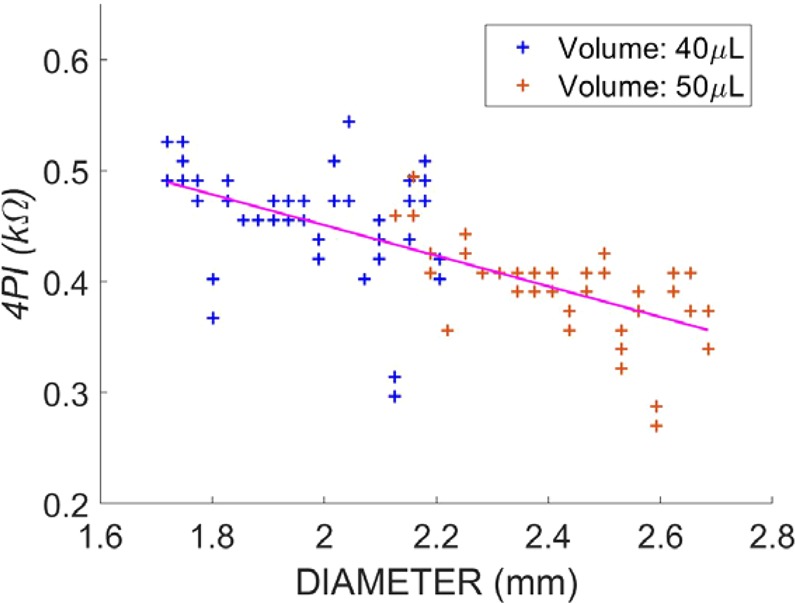
Four-point impedance measurements corresponding to the cross-sectional area from the insertions into the 3D printed cochlea models. All data points are shown along the respecting cross-sectional area dependent on the location of the electrode array within the cochlea model. The regression line is plotted (y = 726 − 138x), with a Pearson’s coefficient of −0.6652.

Having found that the cylindrical diameter was the main determinant of 4PI, the impedance measurements made from the two cochlear models were plotted against the scalar diameter at the point at which the measurement was made (Fig. 6). Impedance decreased linearly with diameter over the range tested, with a 200 Ω difference from 1.7 mm to 2.7 mm. There is some overlap between the two models around the diameter size of 2.2 mm, where the diameter at the basal electrode in the smaller model was similar to that at the apical electrode in the larger model.

In experiments on the 40 µL model in which the electrodes were facing the modiolus, 10 µL of either blood, or an artificial perilymph control solution, was injected after full electrode insertion (Fig. S4). The red arrow indicates the point of injection in reference to the electrode array. Impedances were unaffected by the injection of artificial perilymph into the cochlea (data not shown). Upon the injection of blood, the 4PI rose locally by ≈400 Ω. This pattern of response persisted for the 2 minutes over which recordings were made.

## Discussion

Higher 4PIs were associated with a total loss of residual hearing three months post-insertion. All patients with high 4PIs (as defined by maximal efficiency on the ROC) also exhibited fluctuation in, or a permanent drop in, the CM amplitude during implantation. The disturbance in CM amplitude implies that there has been some interference with normal cochlear function, as may be caused by contact between the electrode and an intracochlear structure critical to hearing. For a straight cochlear implant electrode array, the vulnerable structures are the lateral cochlear wall and the basilar membrane^16^. All straight electrodes come into contact with one or both of these structures if inserted deeply enough^17^. Contact between the electrode and the basilar membrane will interfere with cochlear mechanics and cause a hearing loss, while trauma (rather than contact) with the lateral cochlear wall is thought to cause hearing loss through disruption of cochlear ionic homeostasis. It is thought that a disturbance in CM amplitude during electrode insertion reflects one of these electrode-cochlear interactions^7,8,18,19^. Contact between the electrode and the endosteum (or in the case of the basilar membrane, epithelium) lining scala tympani is implicit in either of these interactions. The increase in 4PI suggests an alteration in the electrical properties of the fluid in the immediate vicinity of the implant’s electrodes, and in the context of surgery, intracochlear bleeding, caused by trauma to a vein or capillary within the endosteum/epithelium, would be the likely candidate. The lateral cochlear wall, with which the straight electrode interacts, is known to have a rich blood supply, and the risk of bleeding during implantation has been reported previously in anatomical studies^20^. Furthermore, bleeding from discrete vessels has been observed on histology performed immediately after experimental cochlear surgery^21^. Therefore, cause of high 4PI during surgery may be acute intracochlear bleeding. If so, the total loss of hearing and post-operative dizziness would not be surprising, given the known downstream effects of blood within the cochlea^20^, as discussed below. We consider more closely the evidence in support of this explanation, and the alternative that high 4PI could relate to cochlear size, geometry or electrode positioning.

The animal study confirmed that 4PI increases instantaneously, and is sustained, when there is blood within the cochlea. The rise in 4PI after the introduction of blood within the guinea pig cochlea is similar to the difference between the low and high 4PI’s observed in patients. The transient spike upon introduction of cold blood into the cochlea was likely a temperature-mediated effect, given that temperature and impedance are inversely related^22^. The baseline impedance values were higher in the guinea pig than the clinical data. Our *in-vitro* studies suggest that this could be accounted for by the smaller size of the rodent cochlea.

A potential cause of high 4PI observed in some patients was cochlear size. We have shown here that it is the diameter of a cylinder, not its’ volume, that is the primary determinant of 4PI when filled with isotonic fluid. This we attribute to a reduction in the availability of conductive ions in the vicinity of the electrodes as diameter decreases. Within the cochlea, there is a reduction in diameter of cochlear scalae from base to apex. In both the *in-vitro* cochlear models and in the clinical data, we observed a gradual increase in 4PI towards the apical cochlear electrodes. But the magnitude of this gradual rise (~30 Ωs) was not large enough to reproduce the high 4PI’s observed in some of the patients, even when the electrode array was inserted into a small volume cochlear model. Similarly, as the cochlea tends to narrow with increasing depth, it was conceivable that the high 4PI patients may have substantially deeper insertions than the low 4PI patients, however the comparable measured angular insertion depth between the groups does not support this interpretation. These observations suggest that cochlear size cannot account for the high 4PIs observed in the patients.

Another potential explanation for the high 4PI was close contact between the electrode array’s contacts and the high-electrical-resistance lateral cochlear wall. In fact, the pattern of 4PI across the electrodes observed in patients was replicated *in-vitro*, by inserting the electrode array backwards, so that the electrical contacts were facing, and in close proximity to, the lateral cochlear wall. However, this cannot explain the results observed clinically, because anti-modiolar rotation of the electrode was infrequent and no more so in the low than the high 4PI group. For this reason, electrode-cochlear wall contact is unlikely to explain the current clinical findings.

The evidence presented here supports the interpretation that intra-cochlear bleeding, caused by trauma to the blood vessels within the cochlear endosteum/endothelium of the lateral cochlear wall, is the most likely explanation for the high 4PI observed clinically. Consistent with this, high 4PI was seen most often on electrodes in the apical half of the array, corresponding to the cochlear region where the array expected to contact the lateral wall^14^. It is also of note that the force required to insert a cochlear electrode increases exponentially with the depth of insertion^10^, so the risk of trauma to the endosteum will increase accordingly. The rapidity of development of the high 4PI also suggests bleeding. While it is conceivable that a transudation of proteins from serum into perilymph may occur acutely after CI, through the increase in vascular permeability associated with surgical wounding, this would be expected to develop over several hours, and not to have influenced the immediate post-insertion impedance measurements reported here. The presence of blood within the cochlea is thought to initiate an inflammatory cascade that is detrimental to hearing. In experimental settings, the injection of blood into the guinea pig cochlea results in cochlear inflammation, a significant elevation in auditory brainstem response thresholds^10,11^, and the development of extensive fibrosis and ossification^15^. These observations may provide a basis for understanding why high 4PI was associated with a total loss of residual hearing. Similarly, high rates of disequilibrium were encountered in patients with high 4PI, and this too may have been a consequence of greater labyrinthine inflammation^23^ associated with bleeding.

We saw perturbations in cochlear microphonic amplitude during intra-operative ECochG that were not associated with high 4PI. This may have occurred if the electrode came into contact with the basilar membrane, dampened its movement and thus decreased cochlear sensitivity^7,17,24^, without causing an overt bleed. Recording both ECochG and 4PI provides different dimensions of information, that together provide greater insight into cochlear status during implantation.

This study demonstrates that a type of electrical impedance might act as a biomarker for the detection of changes in the electrical environment within the cochlea during cochlear implantation (most likely caused by bleeding) that relate to clinically significant outcomes, such as hearing preservation and dizziness. We have shown that 4PI has the sensitivity to detect changes that the conventional impedance measurements made by commercial cochlear implant software (such as common ground impedance) cannot. This finding adds a new dimension to intracochlear monitoring, where electrophysiological monitoring with ECochG has recently emerged as a successful biomarker for monitoring residual hearing, hair cell or neural function and insertion trauma, which has enabled the prediction of post-operative hearing loss and CI function^25–27^. Here we demonstrate that intra-operative, intracochlear monitoring with both ECochG and 4PI allows a deeper understanding of the likely cochlear response to implantation than either alone. The new information provided by 4PI allows further elucidation of the likely mechanisms of cochlear trauma and its sequelae such as hearing loss, that may open up new possibilities for surgical intervention to minimise loss of cochlear function during inner ear surgery.

Intraoperative monitoring is transforming the possibilities for the treatment of hearing loss. These tools are increasing the likelihood that hearing will be preserved during cochlear implantation, and this has immediate benefits for recipients, who may be able to take advantage of improved auditory perception through electroacoustic hearing. Such an ear is also likely to have retained sufficient cochlear function and structure to be eligible for future therapies that may regenerate hair cells and/or neurons. Intraoperative monitoring brings us a step closer to a future when patients requiring a cochlear implant will retain their residual hearing and be candidates for future regenerative therapies.

The coarse temporal resolution of the audiology and the 4PI measurement in this study has prevented analysis of the time-course of the association between the two, but it is apparent that intraoperative events do predict a later loss of hearing. While we gain some further insights concerning 4PI and auditory function from intraoperative ECochG, more frequent hearing assessments would determine whether the hearing loss is immediate or delayed, and this would further inform the underlying aetiology of high 4PIs and the subsequent cochlear response.

A limitation of the clinical data available to date is that for technical reasons 4PI could not be inter-leaved with the ECochG recordings. This meant that 4PI was recorded after complete electrode insertion, and it was not possible to observe the dynamics of its’ elevation during the implantation procedure. Such observations would likely add further weight supporting either intracochlear bleeding or potentially electrode cochlear shape/electrode orientation as the critical determinant of a high 4PI measurement.

The *in-vitro* models studied here were fabricated from a “perfect” electrical insulator. This does not reflect the situation in the cochleae of mammals. While it is accepted that the lateral cochlear wall has a very high impedance^28^ the impedance of the medial cochlear wall is lower, given that it more porous. This difference between the model and real cochleae does not affect the main outcome of the *in-vitro* studies, that cochlear size and scalar diameter have an influence on 4PI, but these trends may be less clear *in vivo*, due to the lower resistance pathways that exist in life. The near-perfect isolation of the model cochlear may have accentuated the effect on 4PI of placing cochlear electrodes in close proximity to the lateral cochlear wall. Another consideration is that the cochlear models were “idealised”, based upon a mathematical model that assumes that scalar shape does not change from cochlear base of apex. This is an approximation to real cochlear anatomy, but one that was unlikely to have affected the *in-vitro* results.

Finally, here was no partitioning of the cochlear scalae in the cochlear models. The absence of a basilar membrane wasn’t expected to have impacted upon the electrical impedance of the cochlear model^29^, but it may have influenced the positioning of the electrodes along the lateral cochlear wall, which in life is impeded by the basilar membrane. However, this was unlikely to have affected our conclusion that 4PI is high when electrode contacts come into close proximity with the lateral cochlear wall.

## Methods

### Clinical devices and study design

The clinical research was carried out in accordance with the relevant guidelines and regulations of the Human Research and Ethics Committee of the Royal Victorian Eye and Ear Hospital Human Research Ethics Committee (approved under Project #14/1171H), who approved all protocols. The conduct of the study conformed in all respects to the Australian Government’s National Statement on Ethical Conduct in Human Research (2018). All operations were undertaken at the Royal Victorian Eye and Ear Hospital between January 2016 and June 2018. Written informed consent was obtained for all participants.

The subjects were adult cochlear implant candidates with pre-operative audiometric thresholds of 85 dB hearing level (HL) or better at 0.25 and 0.5 kHz. These patients received either the Cochlear CI422 or CI522 Nucleus™ cochlear implants. Both implants have the same Slim Straight™ array, which includes 22 half-band intra-cochlear electrodes over 20 mm (Cochlear Ltd., Sydney, Australia). All electrodes were implanted to a depth of 20–25 mm (at the surgeon’s discretion) via a round window approach. The speed of electrode insertion was not prescribed and ranged between 1–2 minutes. The cochleostomy was sealed with a thin piece of fascia. As part of routine intraoperative care, patients received dexamethasone 10 mg i.v. after induction of anaesthesia.

During surgery, ECochG was recorded while the electrode array was implanted into the cochlea, and the electrode leads were secured within the mastoid cavity. Four-point impedance was measured once the ECochG had been completed. These procedures were video-recorded from the operating microscope, and later reviewed by an independent surgeon to determine the orientation of the electrode array during its insertion into the cochlea. Following surgery, the implanted cochlea was imaged in Stenver’s View, from either a plain radiograph or a CT scan, and the angle of insertion was derived.

Audiometry was repeated 3 months following surgery. Total hearing loss was defined as ≥110 dB HL at 0.25 and 0.5 kHz, ≥115 dB HL at 1 kHz and ≥120 at 2, 4 and 8 kHz. When residual hearing was better than these levels, the hearing loss after surgery was calculated by subtraction of the pre-operative from the 3-month audiograms on a frequency-by-frequency basis.

Medical records were reviewed to identify episodes of post-operative dizziness within three months of implantation. To ensure that the dizziness reported here was a post-operative event and not a pre-existing problem, the entire medical record was reviewed, with particular reference to the general medical history, pre-operative otological history and post-operative follow up.

### Four-point impedance recordings

4PIs were measured using in-house custom written software to control Cochlear’s Nucleus™ cochlear implants’ inbuilt voltage measurement function, accessed through the Cochlear Device Interface (CDI) libraries (4.15.02). The measurements are taken as instantaneous voltage values derived from the DC component of the applied current. Due to nature of the instantaneous measurement, the impedance derived contains only resistive information and therefore no capacitive or inductive components.

4PIs were obtained from 4 adjacent electrodes. The outer two electrodes were the source and the return for current stimulation. Stimulation was a charge-balanced biphasic pulse, with pulses of 25 µs in length and a 7.5 µs interphase gap. A single voltage measurement was taken at the end of the first phase of the biphasic pulse from the central pair of electrodes, using the inbuilt voltage measurement function of the implant. The measurements were streamed to an external sound processor, connected in turn to a Freedom™ programming device (POD) interfaced by USB with a PC laptop (Dell, TX, U.S.A.).

In the clinical studies, 4PI was recorded from 19 sets of 4 consecutive electrodes, with the applied current (to outer two of the four electrodes) at 120 current levels (approx. 0.11 mA). Receiver-operator curves (ROCs) calculated the sensitivity and specificity of different 4PI cut-offs predicting a total hearing loss three months later. If the area-under-the-curve was significantly above 0.5, then resampling techniques were used to determine statistics of the point of “maximum efficiency”, positive (PPV) and negative (NPV) predictive values using 1000 bootstrap replicas. 4PI exceeding the point of maximum threshold were defined as “elevated” or “high”.

### Common ground impedances (CGIs)

CGIs were taken as part of routine clinical practice at the Royal Victorian Eye and Ear Hospital’s Cochlear Implant Clinic using Custom Sound EP’s “Measure Impedances” function. Impedances were routinely measured on the day of implantation using Cochlear’s NRT tool.

### Electrocochleography (ECochG) recording and analysis

ECochG was recorded with our custom Cochlear Response Telemetry (CRT) system, as described previously^7^. Briefly, CRT records cochlear potentials evoked by acoustic stimulation of the cochlea. The system uses Cochlear’s Nucleus™ NRT™ amplifier to record (at a sampling rate of 20 kHz) the cochlear potentials from any of the intracochlear electrodes, using the extra-cochlear plate electrode located on the body of the implant as a reference. ECochG was recorded intra-operatively while the CI’s electrode array was implanted into the cochlea, and its leads stabilised within the mastoid cavity.

Acoustic stimuli were generated by a USB digital stimulation and acquisition card at 192 kHz (DT9847, Data Translation, MA, USA) and presented closed-field to the ear canal via an ER3A insert phone (Etymotics, IL, USA). The acoustic output of the ER3A was calibrated to the dB HL scale, using a GRAS 43AG artificial ear (G.R.A.S Sound & Vibration A/S, Denmark), a Norsonic NOR140 sound level meter (Norsonic AS, Norway), and an oscilloscope (DS1102E, Rigol Technologies Inc, OR, U.S.A). All acoustic stimuli were presented with alternating condensation and rarefaction polarities, and the responses saved in separate buffers for later analysis.

During the insertion of the cochlear implant, ECochG was recorded over 12-ms from the most apical electrode of the array. The acoustic stimulus was a 0.5 kHz tone burst of 6-ms duration with 1-ms rise/fall times, presented 14 times per second at 100 dB HL. These were observational recordings, where the CRT operator provided no feedback to the surgeon during the procedure.

The ECochG was processed by taking the difference of the rarefaction- and condensation phase-responses and dividing this by two (the “DIF” waveform). From this response, the cochlear microphonic (CM), which primarily reflects outer hair cell activity^7,8,19,25^, can be derived by applying a 15th order Hamming window from 0.9X to 1.1X the stimulus frequency.

The criterion for the detection of potential hearing perturbation (possibly indicative of cochlear injury) during implantation was the behaviour of the CM amplitude during insertion of the electrode into the cochlea. “CM-drop” insertions, were defined as those with a CM amplitude drop of >30% from preceding values at any point of time during the insertion of the electrode, during the placement of the electrode lead in the mastoid cavity, or when sealing the round window with fascia^7^.

### Animal experimental methods

All methods were carried out in accordance with the guidelines and regulations of the *Australian code for the care and use of animals for scientific purposes, 8*^*th*^ *ed*, published by the National Health & Medical Research Council of Australia. All experimental protocols were approved by the Animal Research Ethics Committee of the Royal Victorian Eye and Ear Hospital (Project #17/375AU). Adult Dunkin-Hartley tricolour guinea pigs, weighing >700 g, underwent left and/or right sided cochlear implantation, all performed by the one surgeon (CS). All animal procedures were performed under anaesthesia of ketamine (60 mg/kg i.m., Troy Laboratories Pty, Ltd, Sydney, Australia) and xylazine (4 mg/kg i.m., Troy Laboratories Pty Ltd, Sydney, Australia). A local anaesthetic, lignocaine (1 mg/mL, Troy Laboratories Pty Ltd), was injected subcutaneously prior to the first incision and throughout the procedure.

Four-point impedance measurements (using the method described above) were made from 13 ears of 9 guinea pigs before and after blood was injected into the inner ear. For these experiments, a custom cochlear electrode array was built consisting of 4 half-band (0.3 mm by 0.3 mm) intra-cochlear electrodes over 3 mm, and two extra-cochlear bullet-shaped electrodes. With this electrode, one configuration was available for the 4PI measurements. The stimulus parameters and acquisition system were identical to those used for the human study.

The surgical approach was via a post-auricular incision. The soft-tissues were dissected down to the bulla and an occipital vein was exposed for blood collection. The extra-cochlear bullet electrodes were placed subcutaneously and a bullostomy was performed with a 1.8 mm cutting burr. Cochlear implantation was performed via a cochleostomy, created with a 0.8 mm diamond burr, located anteroinferiorly to the round window in order to avoid injury to the basilar membrane^10^. Care was taken to avoid overt bleeding at the site of the cochleostomy. The electrode was inserted, avoiding resistance, to a depth of 3 mm. After measuring 4PI for at least five minutes, blood was injected into the inner ear with a 30-gauge needle attached to a syringe, placing approximately 0.3 mL of blood into the cochleostomy. In most cases, the blood was freshly drawn and of body temperate. In several cases, the blood had been collected earlier and placed on ice to prevent clotting. Post-blood injection, 4PI was recorded for approx. 10 minutes. Control animals were implanted and monitored, with no attempt made to inject into the cochleostomy.

After the experimental procedures, the animals were euthanised with a lethal dose of pentobarbitone (2.5 mL). Transcardiac perfusion was performed using 0.9% phosphate-buffered saline followed with 10% neutral buffered formalin, and the cochleae were removed and placed in 10% neutral buffered formalin, taking care to not disturb any blood around the cochleostomy. Cochleae were decalcified in 10% ethylenediaminetetra-acetic acid (EDTA) for five weeks, washed with 10%-15% sucrose and embedded in Aliquot Optimal Cutting Temperature (OCT by Tissue Tek, Sakura Finetek USA Inc, CA, USA). Once frozen, 10 µm thick mid-modiolar cochlear sections were cut on a cryostat. The sections were then dried and stained for histological analysis with a Haematoxylin (Mayers Haematoxylin) and Eosin (Putts Eosin) stain and captured on a Ziess Axioplan 2 microscope with AxioVision Software (ZEISS, Oberkochen, Germany).

### *In-vitro* studies

*In-vitro* experiments were conducted in 3D printed cochlear models and cylinders, to determine how the geometry of the cochlea affected 4PI measurements. To do so, cochlea models were 3D printed using the Formlabs Form 2 printer (Somerville, MA, USA), which uses a stereolithography technique where resin is solidified via a laser. The resolution in the z-direction was 50 µm using Formlabs clear photopolymer resin.

The cochlea models were derived from parameters of the scala spine and cross-sectional areas previously used in studies, representing the whole cochlea size, producing a 50 µL model which was then scaled to create a 40 µL model^30,31^. These models reflected human total cochlear volume but did not implement a structure for the basilar membrane as there were no barriers between the scalae, allowing the electrode array to be positioned anywhere in the model. Models were filled with artificial perilymph^32^ (containing NaCl 125, KCl 3.5, NaHCO3 25, MgCl2 1.2, CaCl2 1.3, NaH2PO4 0.75, C6H12O6 5.0) to emulate the conductive fluid seen in human cochleae.

To further explore geometric parameters influencing 4PI, another series of cylinders were 3D printed into resin, using the same printer and parameters previously described. Two sets of cylinders were produced, one comprising of different volumes, ranged from 30 to 80 µL while the diameter remained constant at 2.2 mm, and the other had a constant volume of 60 µL while the diameter varied between 1.6 to 2.8 mm. These diameters replicated those diameters encountered in the cross-sectional areas for the 3D printed cochlea models. In this experiment, normal saline was used to fill the cylinders as it has similar conductive properties to artificial perilymph.

The Cochlear Nucleus™ 522 “Slim Straight ™” electrode array was used, as per the clinical studies. The electrode wires were connected to a D25 male plug, which connected directly to a Cochlear Nucleus™ Freedom implant.

The impedance measurements using the methods described for the clinical studies. The 4PI was measured after the electrode array was completed inserted into the models. The electrode array was designed to sit along the lateral wall, with the electrode contacts facing towards the modiolus, however surgeons have been known to rotate the electrode inadvertently which could bring the electrodes into contact with the cochlear walls. To determine whether this might have affected the 4PI measurements, electrodes were inserted into the cochlear model with the electrodes either facing the cochlear modiolus or its’ lateral wall.

The 3D printed cochlea models provide a simplified platform for observing the effect of blood on 4PI, without the variability inherent in biological models. In some experiments on the 40 µL cochlea model, following insertion of the electrode (with contacts facing the modiolus), 10 µL of human blood was injected via a hole drilled at the region where the basal turn first begins to curve in the model. 4PI was recorded before and after blood injection. For these experiments, fresh human whole blood was stored in a vacutainer tube, at room temperature, with ethylenediaminetetraacetic acid to ensure that it did not clot before injection (Human Research Ethics Committee of the Royal Victorian Eye and Ear Hospital, Project #18/1396 H).

## Supplementary information

Supplementary information.
