# Supplementary material for: Four-point impedance as a biomarker for bleeding during cochlear implantation
Source: Sci Rep. 2020 Feb 17;10:2777. doi: 10.1038/s41598-019-56253-w (PMC7026160; doi:10.1038/s41598-019-56253-w)
Supplement: Supplementary file 1 — Supplementary information. [file 41598_2019_56253_MOESM1_ESM.docx]

Supplementary Materials for

**Four-point impedance as a biomarker for bleeding during cochlear implantation**

Christofer Bester1¶, Tayla Razmovski1¶, Aaron Collins1, Olivia Mejia, Søren Foghsgaard, Alistair Mitchell-Innes, Chanan Shaul1, Luke Campbell1, Hayden Eastwood1, Stephen O’Leary1,2*

Correspondence to: sjoleary@unimelb.edu.au

**This PDF file includes:**

Figs. S1 to S4

Fig. S1.

Average 4PI profile of insertions from patients with low 4PI. The average 4PI across the electrode array derived from the 38 patients with ‘low’ 4PI, immediately after implantation.


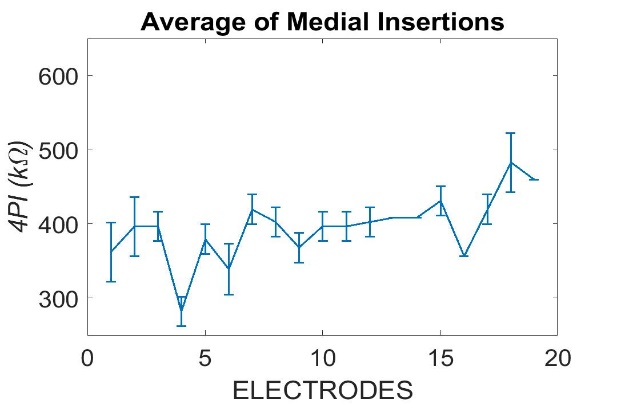

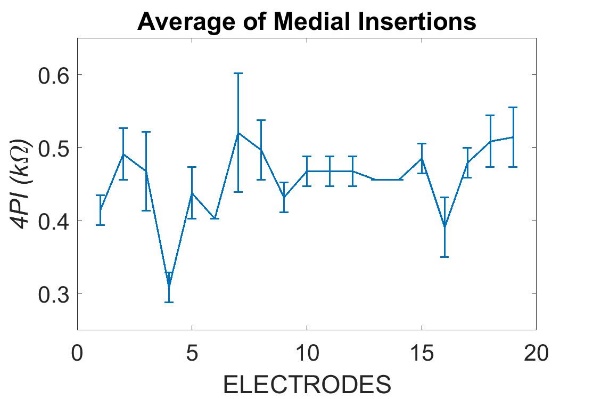

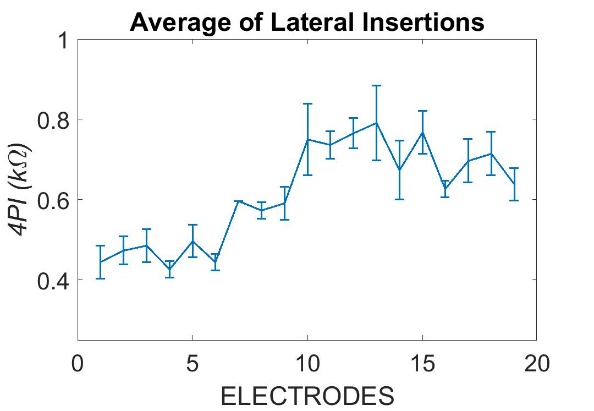

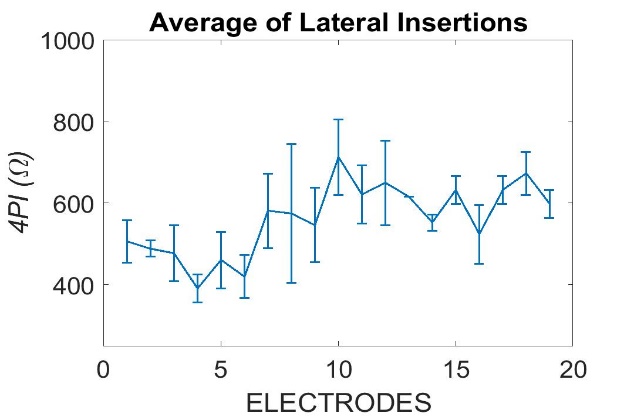


Volume: 50μL

Volume: 40μL

**A**

**C**

**D**

**B**

Fig. S2.

Average 4PI profiles for insertions into both 3D printed cochlea models. **A)** and **C)** show the average 4PI when the electrode array was implanted into the cochlea model that has a net volume of 50µL while **B)** and **D)** show the average for the 40µL model. **A)** and **B)** display the 4PI when the electrodes are facing towards the modiolus of the cochlea, and **C)** and **D)** are the profiles for when the electrodes are facing the lateral wall of the model.


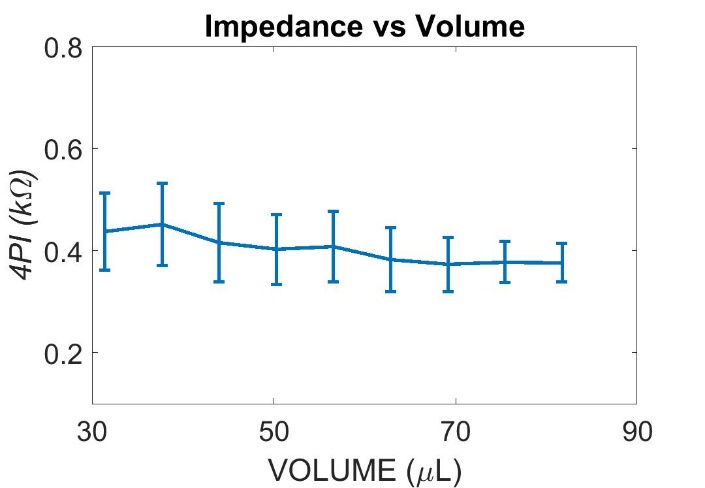

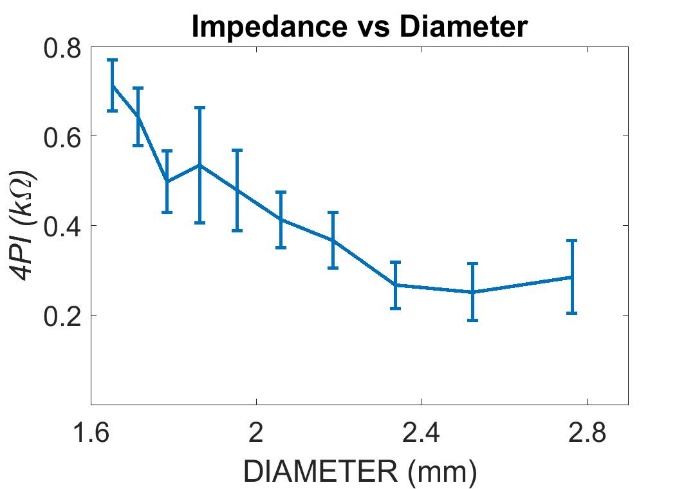


**A**

**B**

Fig. S3.

Average four-point impedance (4PI) from the tests involving the cylinders with either varying diameter or volume. **A)** Results from the cylinders that had a diameter of 2.2mm but with different volumes (lengths). **B)** Displays the 4PI obtained from cylinders having a 60µL volume but with different diameters.


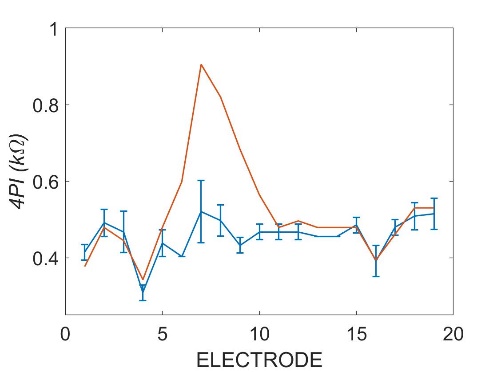


**Fig. S4.**

Four-point impedance measurements compared when blood was injected into the 3D printed cochlea model, with the average measurements from the insertions. The red arrow indicates the point of blood injection into the cochlea model with respect to the electrode array.
